# Breast cancer diagnosed during pregnancy is associated with enrichment of non-silent mutations, mismatch repair deficiency signature and mucin mutations

**DOI:** 10.1038/s41523-018-0077-3

**Published:** 2018-08-06

**Authors:** Bastien Nguyen, David Venet, Hatem A. Azim, David Brown, Christine Desmedt, Matteo Lambertini, Samira Majjaj, Giancarlo Pruneri, Fedro Peccatori, Martine Piccart, Françoise Rothé, Christos Sotiriou

**Affiliations:** 1 Breast Cancer Translational Research Laboratory J.-C. Heuson, Institut Jules Bordet, Université Libre de Bruxelles (ULB), Brussels, Belgium; 2 Department of Internal Medicine, American University of Beirut (AUB), Beirut, Lebanon; 3 Department of Pathology, Memorial Sloan Kettering Cancer Center, New York, USA; 4 Department of Pathology, IRCCS Istituto Nazionale Tumori, Milan and University of Milan, School of Medicine, Milan, Italy; 5 European Institute of Oncology, Milan, Italy

## Abstract

Breast cancer diagnosed during pregnancy (BCP) is a rare and highly challenging disease. To investigate the impact of pregnancy on the biology of breast cancer, we conducted a comparative analysis of a cohort of BCP patients and non-pregnant control patients by integrating gene expression, copy number alterations and whole genome sequencing data. We showed that BCP exhibit unique molecular characteristics including an enrichment of non-silent mutations, a higher frequency of mutations in mucin gene family and an enrichment of mismatch repair deficiency mutational signature. This provides important insights into the biology of BCP and suggests that these features may be implicated in promoting tumor progression during pregnancy. In addition, it provides an unprecedented resource for further understanding the biology of breast cancer in young women and how pregnancy could modulate tumor biology.

## Genetics: pregnancy-associated breast cancer shows altered mutational landscape

Breast cancers diagnosed during pregnancy exhibit unique molecular characteristics that could explain their aggressive nature. Christos Sotiriou from the Jules Bordet Institute in Brussels, Belgium, and colleagues compared the molecular and genetic profiles of tumors from women diagnosed while pregnant with those from age-matched, non-pregnant controls. Although genome-wide copy number alterations were similar in the two groups, tumors from patients diagnosed during pregnancy harbored a greater number of protein-altering gene mutations on average. Mutations were especially common in genes encoding mucins, a group of proteins involved in cancer progression. Pregnant patients were also associated with a mutational signature linked to mismatch repair deficiency. These results point to ways in which pregnancy may modulate tumor biology. The high mutational load found in women diagnosed during pregnancy also suggests these patients may be good candidates for immunotherapy.

## Introduction

Breast cancer is the most frequently diagnosed malignancy during pregnancy.^1^ Its incidence is increasing given the rising trend of delayed childbearing.^2^ Given its rarity, few dedicated studies were performed so far; hence, our understanding of these tumors remains poor. The clinical management of these patients follows standard guidelines with only minor adaptations according to gestational age, maternal wishes and fetal considerations.^2^ Therefore, the molecular characterization of BCP goes beyond academic curiosity as it is of utmost clinical interest to determine if these patients should be treated similarly to non-pregnant breast cancer patients. In this report, we aimed to identify specific molecular alterations characterizing BCP by combining whole genome sequencing, copy number alteration and gene expression data.

## Results

A total of 167 patients with primary breast cancer were retrospectively included in this study, 54 of whom were diagnosed during pregnancy. Detailed patient characteristics were previously published.^3^ At a median follow-up of 9 years, median disease-free survival (DFS) time of BCP was 9.8 years vs. 12.5 years in controls (*P* *=* 0.041, log rank test, Supplementary Fig. S1a). Observed 5-year overall survival (OS) rate was 95.5% vs. 85.1% in BCP and control, respectively; median OS time was not reached within the time frame of the study (Supplementary Fig. S1b). In a multivariable Cox proportional hazards regression of DFS and OS, adjusted for age at diagnosis, date of diagnosis, pathological stage and molecular subtypes by IHC, we found that BCP was associated with worse DFS (multivariable hazard ratio [mHR] 1.81; 95% CI 1.09–3.01, *P* = 0.024) and OS (mHR 2.53; 95% CI 1.20–5.36, *P* = 0.017) (detailed survival data is provided in Supplementary Table S1).

### BCP and controls have similar somatic copy number alteration profiles

**Fig. 1:**
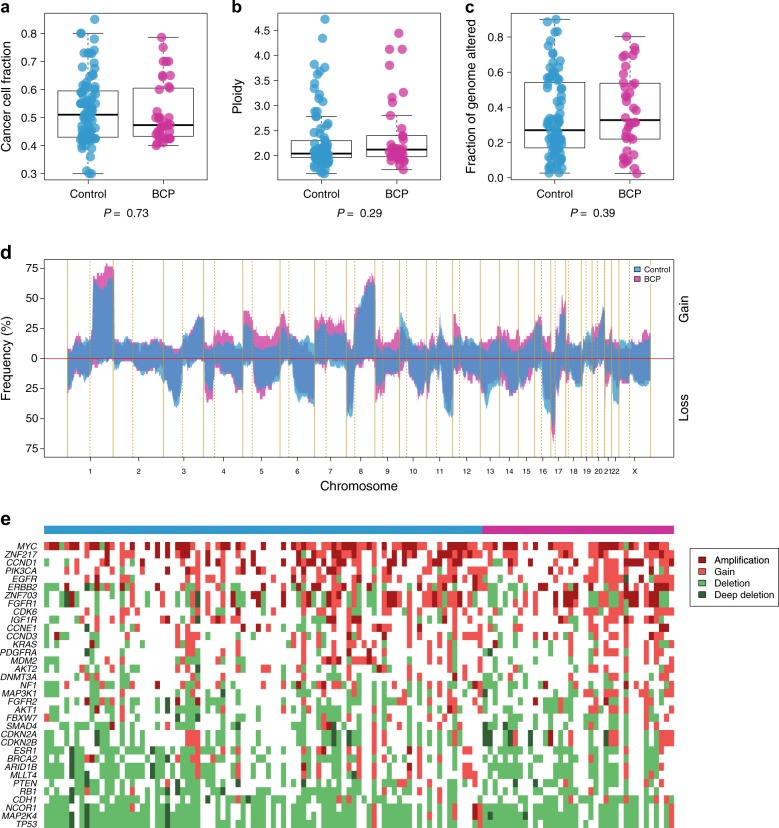
Summary of the genome-wide copy number analysis of 87 controls and 38 BCP tumor samples. **a**–**c** Comparison of cancer cell fraction, ploidy and fraction of genome altered between controls and BCP. **d** Comparison of the CNA frequencies of controls (blue) and BCP (pink). **e** Heatmap of 35 CNA breast cancer driver genes according to their alterations; controls (blue) and BCP (pink). *P*, *p*-value derived for the Mann–Whitney *U* test

We first sought to investigate whether tumors from BCP patients show distinct copy number alterations (CNAs) compared to tumors from matched non-pregnant breast cancer patients (controls). Hence, we performed genome-wide copy number alterations profiling on 160 formalin-fixed paraffin-embedded (FFPE) primary tumor samples from 52 BCP patients and 108 controls. Of note, gene expression data were available for all patients as previously described.^4^ After quality control, CNA profiles were obtained for 125 tumor samples (78%) from 38 BCP and 87 controls. The main reason for exclusion was low cancer cell fraction (CCF < 30%) as estimated with the Genome Alteration Print algorithm^5^ (Supplementary Fig. S2). No differences in clinicopathological features were observed between BCP and controls (Supplementary Table S2). We found no significant differences between BCP and controls in terms of cancer cell fraction, ploidy, and fraction of genome altered (Fig. 1a-c). Moreover, no significant differences were observed between the CNA profiles of the two groups neither at the segment nor at the chromosome arm levels, including the gains of 1q and 8q and loss of 8p, reported to frequently occur in breast cancer^6^ (Fig. 1d). We also compared CNAs profiles by intrinsic subtypes as defined by PAM50 and found no significant differences (Supplementary Fig. S3 and Supplementary Table S3).

We next focused our analysis on the 35 genes that were previously identified as CNA drivers in breast cancer.^7^ As expected, *MYC* oncogene was the most frequently gained/amplified whereas *TP53* tumor suppressor gene was the most frequently lost/deleted across the whole cohort (Fig. 1e). Using GISTIC2.0,^8^ we identified 22 focal amplifications and 23 focal deletions and found no differences between their prevalence in the two groups (Supplementary Fig. S4). Taken together, these results suggest that the CNA profiles of BCP and controls are similar.

### BCP shows a higher number of non-silent mutations

**Fig. 2:**
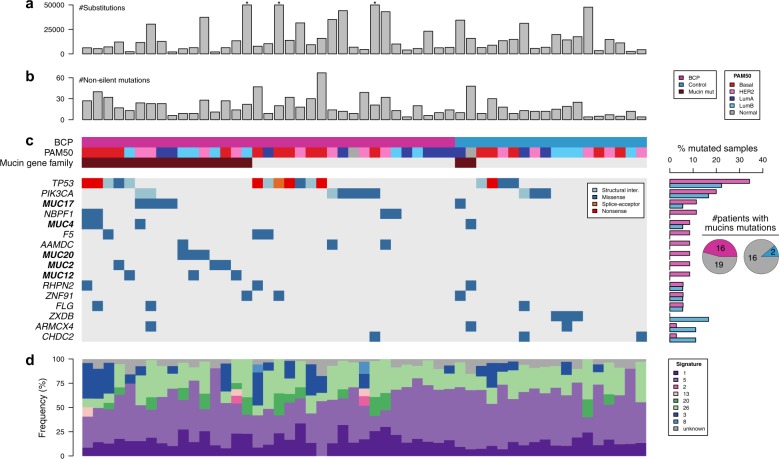
Mutational landscape of individual BCP and controls. **a** Bar chart representing the absolute number of substitutions in BCP and controls, y-axis limited to 50,000 indicated by (*). **b** Bar chart representing the absolute number of non-silent mutations in BCP and controls (median: 20 vs. 12, *P* = 0.027, respectively). **c** Co-mutation plot showing genes harboring at least one non-silent mutation with a frequency of at least 5% across the whole cohort, and their corresponding frequencies in BCP and controls (right). **d** Proportion of breast cancer substitution signatures in each sample. Signatures are colored according to broad biological groups: 1 and 5 are associated with clock-like processes, 2 and 13 are APOBEC-related, 20 and 26 are associated with mismatch-repair deficiency, 3 and 8 are associated with homologous-recombination deficiency

To identify potential genomic differences between BCP patients and controls, we performed whole genome sequencing (WGS) on paired DNA samples extracted from FFPE blocks (i.e., primary tumors and histologically normal axillary lymph nodes) in a subset of 53 breast cancer patients from our initial series, 35 of whom were BCP (Supplementary Fig. S2 and Supplementary Table S2). We achieved 32X and 19X median haploid genome coverage for tumor and normal samples respectively, which is similar in range to previous studies^7^ (Supplementary Fig. S5). We detected a median of 13,829 and 10,084 single nucleotide variants (SNVs) and a median of 21 and 26 small insertions and deletions (Indels) in BCP and controls, respectively, and found no difference between the two groups (Fig. 2a and Supplementary Fig. S6a-c). Moreover, there was no difference in structural variations (insertions, deletions, duplications) nor tumor heterogeneity as assessed by the MATH score^9^ (Supplementary Fig. S6d-f).

We identified a median of 14 non-silent mutations per tumor which is comparable to another large-scale breast cancer cohort study^7^ (Supplementary Table S4). Interestingly, BCP had a significantly higher number of non-silent mutations than controls (median: 20 vs. 12, *P* = 0.027, Fig. 2b and Supplementary Fig. S6g-h). This observation remained consistent after correcting for potential confounding factors including age at diagnosis, date of diagnosis, pathological stage and molecular subtypes by IHC (*P* = 0.019, Fig. S6g). Compared to controls, BCP had also a significantly higher number of mutations previously reported in breast cancer in the Catalog of Somatic Mutations in Cancer (COSMIC) database^10^ (*P* = 0.018, Supplementary Fig. S6i). At the gene level, we identified 17 genes harboring at least one non-silent mutation with a frequency of at least 5% across all patients. Of those, *TP53* and *PIK3CA* were the most frequently mutated genes without any significant difference between the two groups (Fig. 2c).

### BCP is associated with a higher frequency of mutations in mucin gene family

*MUC17* was the third most mutated gene and four other mucin gene family members namely *MUC2*, *MUC4*, *MUC12*, and *MUC20*, were among the most frequently mutated genes in BCP (Fig. 2c). Within the mucin gene family, we identified 20 missense mutations and one nonsense mutation in BCP compared to only two missense mutations in controls. Among these 20 mucin variants, 10 were present in the COSMIC database,^10^ which was higher than expected by chance (*P* = 0.006, Monte-Carlo test, Supplementary Table S4). Altogether, we found a significantly higher number of BCP with non-silent mutations in the mucin gene family compared to controls (45.7 vs. 11.1% respectively, *P* = 0.015, Fig. 2c). This observation remained consistent after correcting for classical clinicopathological features (*P* = 0.008). Similar findings were observed by comparing BCP with 56 matched controls taken from the TCGA dataset (45.7 vs. 23.1% respectively, *P* = 0.034). Acknowledging that some mucins (*MUC4, MUC16*) are known to give rise to false positive calls due to technical artifacts,^11^ we removed these two genes and confirmed the above-mentioned results (37.1 vs. 5.5%, *P* = 0.020 and 37.1 vs. 14.3%, *P* = 0.020, using controls and TCGA controls, respectively).

**Fig. 3:**
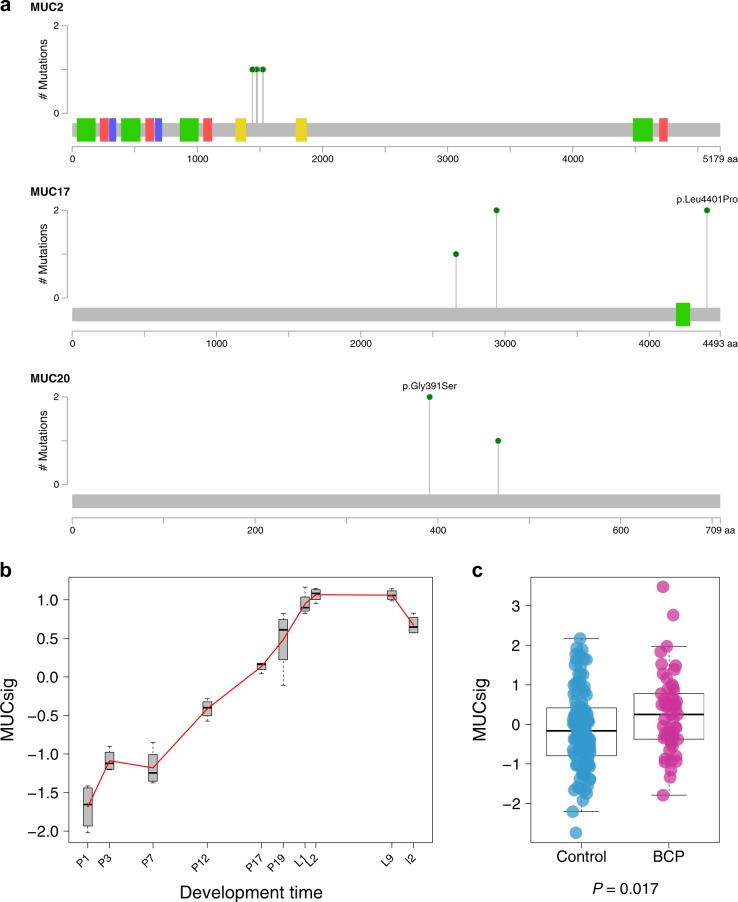
Enrichment of mucin mutations and upregulation in BCP. **a** Lollipop plots were generated using cBioPortal Mutation Mapper. Each lollipop denotes a unique missense mutation for *MUC2*, *MUC17*, and *MUC20* in BCP. **b** MUCsig according to normal adult mouse mammary development (from pregnancy day 1 to involution day 2). **c** Comparison of MUCsig between controls and BCP. *P*, *p*-value derived for the Mann–Whitney *U* test

We did not find any differences in clinicopathological features or survival according to mucin mutational status (Supplementary Table S5 and Supplementary Fig. S7). There were three hotspots mutations (i.e., present in two distinct patients) two in *MUC17* and one in *MUC20*, and five missense mutations were clustered within 260 base pairs of *MUC2* (Fig. 3a). None of these mutations were in annotated protein domains. Since the glycosylation of mucins is known to play a major role in producing a chemical barrier at the epithelium of tubular organs for protection and lubrication, we interrogated whether these mutations could affect glycosylation acceptor sites. The mucin O-glycosylation is characterized by the addition of N-acetylgalactosamine (GalNAc) to the hydroxyl group of serine or threonine residues.^12^ Remarkably, 40.9% of missense mutations affecting mucins resulted in an amino acid change to a serine residue, which was significantly higher than expected by chance (*P* = 0.0002, Monte-Carlo test), suggesting mucin hyperglycosylation in BCP. We also found that the frequency of missense mutations resulting in a gain of serine site in mucins in the TCGA dataset was significantly lower compared with BCP (6.3% in TCGA vs. 40.9% in BCP, *P* < 0.001). Since mucins expression is known to increase throughout gestation in mice,^13^ we expected that mucins were also upregulated in BCP. We therefore derived a metagene signature comprising all members of the mucin gene family (called “MUCsig”) from the corresponding gene expression data and found higher expression of MUCsig in BCP than in controls (*P* *=* 0.017, Fig. 3b-c). Altogether, these results show that BCP is associated with an increased expression of mucins as well as a higher frequency of mutations in mucin gene family that may potentially lead to mucin hyperglycosylation.

### BCP is enriched in mutational signature related to mismatch repair deficiency

**Fig. 4:**
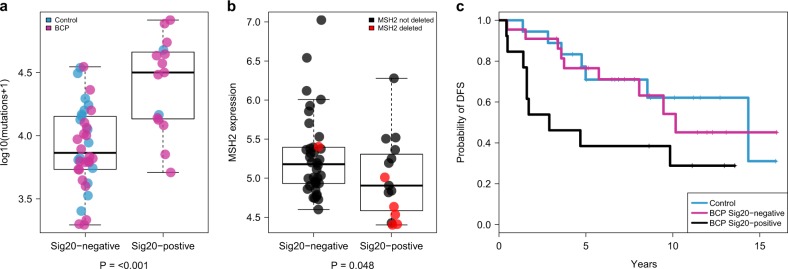
Association of signature 20 with mutational load and clinical outcome. **a** Comparison of SNV mutational load between Sig20 negative and Sig20 positive tumors. **b** Comparison of MSH2 expression between Sig20 negative and Sig20 positive tumors. **c** Kaplan–Meier plot showing the difference in DFS between control patients (*N* = 18), BCP patients with Sig20 negative tumors (*N* = 22) and BCP patients with Sig20 positive tumors (*N* = 13). *P*, *p*-value derived for the Mann–Whitney *U* test

To have a better understanding of the etiology of BCP, we interrogated the contribution of base-substitution signatures known to occur in breast cancer.^7^ When evaluating the proportion of each signature present in each sample, we found that signature 1 was more prevalent in BCP compared to controls whereas signature 5 was more prevalent in controls (*P* = 0.013, FDR *=* 0.053 and *P* = 0.01, FDR = 0.053, respectively, Fig. 2d). These results remained consistent after controlling for clinicopathological features (*P* = 0.002, FDR *=* 0.014 and *P* = 0.004, FDR = 0.016, respectively). When evaluating the presence or absence of mutational signatures we found that signature 20 (Sig20) was found in 13 out of 35 BCP (37.1%), as compared to only 2 out of 18 controls (11.1%) (*P* *=* 0.059, FDR = 0.410, Fig. 2d). When controlling for clinicopathological features, this observation was significant (*P* = 0.004, FDR = 0.029). Signature 1 is known to be associated with age at diagnosis while the etiology of signature 5 is still unclear. Sig20, previously found in stomach and breast cancers, is related to DNA mismatch repair (MMR) deficiency.^14^ Of interest, this signature remained significantly enriched in BCP when increasing the number of controls with 64 matched cases derived from the BRCA560 dataset (37.1 vs. 3.1%, *P* < 0.001). No classical clinicopathological features were associated with BCP Sig20-positive tumors except progesterone receptor negative status (Supplementary Table S6). We found that Sig20 frequency was strongly correlated with SNV mutational load (*ρ* = 0.56, *P* *<* 0.001, Supplementary Fig. S8a) with Sig20-positive tumors harboring a median of 31,632 SNVs, as compared to 7,352 SNVs in Sig20-negative tumors (*P* < 0.001, Fig. 4a). Next, we interrogated if Sig20 could be caused by alteration of genes involved in the MMR machinery either at the expression or copy number levels. The first step of MMR is the recognition of replication errors mediated by MutS homolog complexes; *MSH2* and *MSH6*.^15^ We found a significantly lower expression of *MSH2* in patients harboring Sig20 (*P* *=* 0.047, Fig. 4b) corroborated by a negative correlation between *MSH2* expression and Sig20 frequency (*ρ* = −0.27, *P* *=* 0.024, Supplementary Fig. S8b). This could be partially caused by CNA in *MSH2* since 5 out of 15 Sig20-positive versus 1 out of 38 Sig20-negative tumors harbored *MSH2* deletions (33.3 vs. 2.6%, *P* *=* 0.01). Finally, we interrogated the impact of Sig20 on survival and found that BCP Sig20-positive patients had a shorter DFS than BCP Sig20-negative patients (median DFS time of 2.9 years vs. 10.2 years respectively *P* = 0.091, log rank test, Fig. 4c). In Sig20-positive patients the median OS was 6.72 years while the median OS was not reached in BCP Sig20-negative patients (P = 0.009, log rank test, Supplementary Fig. S9). This was not significant in a multivariate model (DFS mHR 1.06; 95% CI 0.21–4.27, *P* *=* 0.31; OS mHR 0.8; 95% CI 0.12–5.07, *P* *=* 0.81, respectively). Overall, these results suggest that some BCP patients show a defective MMR due to copy number loss of *MSH2*.

## Discussion

This study reveals important molecular differences characterizing BCP that may potentially represent a biologic explanation for their rather aggressive clinical behavior. First, BCP was enriched in non-silent mutations that could have potential oncogenic functiond. Second, 45% of BCP harbored a mutation in mucin gene family in addition to an upregulation of mucins at the mRNA level. Like in mice,^13^ this could be due to physiological change induced by pregnancy to prepare the breast for lactation. Our hypothesis is that some preexisting subclones carrying mucin mutations could have a growth advantage under pregnancy state. Another argument in favor of this hypothesis is the fact that most mucin mutations resulted in an amino acid change to a serine residue and that some of them are in hotspot regions. It has been previously found that in breast cancer, alterations in mucin expression or glycosylation influence tumor growth, adhesion, invasion, and immune surveillance.^16,17^ The impact of missense mutations resulting in an amino acid change to a serine residue on the glycosylation status of mucins is unknown, but it is tempting to speculate that these alterations could influence their function, stability and secretion. More investigations are required to determine the exact effect of mucin mutations in BCP and in breast cancer in general, but these alterations could play a role in BCP biology.

Moreover, BCP showed a higher prevalence of signatures 1 and 20 and a lower prevalence of signature 5. The etiology of signature 5 is not well understood.^18^ The high prevalence of signature 1 cannot be explained by a difference in age at diagnosis or age of the blocks since similar results were found in a multivariate analysis after adjusting for both variables. 37.1% of BCP were associated with signature 20 (Sig20), attributable to DNA mismatch repair deficiency. This is surprising given the low frequency (1–2%) of MMR deficiency recently reported in breast cancer.^19^ Mechanistically, this could be explained in part by the deletion of *MSH2*, a key gene involved in MMR. Survival analysis showed that BCP Sig20-positive patients had the worst prognosis whereas BCP Sig20-negative patients had DFS comparable to controls. MMR deficiency and high mutational burden have been shown to predict clinical benefit to immune checkpoint blockade in colorectal and other types of highly immunogenic cancers.^20,21^ To date, the role of checkpoint inhibitors in the treatment of breast cancer is under intensive investigation and the results are still awaited.^22^ While the feasibility of investigating new agents in such peculiar disease is rather complex, these results could potentially open the door to identify high-risk BCP patients who could benefit from immunotherapy.

A potential limitation of our study is that we used archived FFPE samples that are known to be challenging for WGS due to DNA degradation and induction of artefacts. Indeed, the higher proportion of signature 1 and 5 observed in our study could be due to C > T artefacts induced by formalin fixation. Nonetheless, BCP and controls were processed in the same way with no difference in the age of the blocks and the sequencing coverage reached in normal and in tumor tissues was comparable to other studies.^7^ Another limitation of our study is the lack of epigenetic profiling analysis. As it is known that pregnancy induce epigenetic changes in epithelial cells to support mammary development,^23^ we can hypothesize that these modifications could impact breast cancer biology. Therefore, the study of such modifications in BCP is worthy further investigation. In conclusion, we believe that our work provides important insights into the biology of BCP and a unique resource to study the biology of breast cancer in young women and how pregnancy could modulate tumor biology.

## Methods

### Patients and samples

A total of 167 patients with primary breast cancer were retrospectively included in this study, 54 of whom were diagnosed during pregnancy. All patients were diagnosed and followed up at the European Institute of Oncology (IEO, Milan, Italy) from 1996 to 2010. As previously described,^3^ this is a case-control study, in which pregnant breast cancer patients and controls were matched according to age, tumor size, nodal status, and date of diagnosis. ﻿For the current genomic analysis, we opted to exclude patients who received neoadjuvant therapy to avoid potential impact of treatment on the obtained results. The majority were treated with anthracycline-based regimen (individual patients data are presented in Supplementary Table S1). All patients had available FFPE tissue from the primary tumor resection and there was only one tumor sample per patient. All control patients were pre-menopausal at time of diagnosis. ER/PR-status were defined by ASCO-CAP. For the classification of Luminal A and B we used a cut-off of Ki67 > 20% according to the St Gallen 2015 Consensus Meetings.^24^ Matched normal tissues were collected from histologically confirmed tumor-free axillary lymph nodes or tumor-adjacent normal tissue and there was only one normal sample per patient. FFPE tissue sections were deparaffinized by xylene followed by a 100% ethanol wash. DNA extraction was performed using the QIAamp DNA FFPE Tissue Kit (Qiagen, Hilden, Germany) following the manufacturer’s recommendations. The quantity of double-stranded DNA was evaluated using the Qubit dsDNA BR Assay Kit. For the WGS, we selected 18 control patients based on major clinicopathological features of the 35 BCP patients, namely age at diagnosis, ER status, and grade. All patients provided written informed consent for the use of tissue samples for research purposes as per the IEO institutional policies. This study was approved by the Ethics Committee of Institut Jules Bordet (Number 1782). The validation of the enrichment of mutations in the mucin family genes in BCP were done by comparing the frequency of these mutations in BCP patients with putatively non-pregnant patients retrieved from the TCGA dataset^25^ and selected to have similar age, estrogen receptor (ER) and progesterone receptor (PR) distribution (*N* = 56) (Supplementary Table S7). The validation of the enrichment of signature 20 in BCP were done by comparing the frequency of this signature in BCP patients with putatively non-pregnant patients retrieved from the 560 breast cancer dataset^7^ (referred to as BRCA560) and selected to have similar age, ER and PR distribution (*N* = 64).

### Transcriptomic profiling

All samples were hybridized on Affymetrix Human Genome U219 array plates following the manufacturer’s protocol, as described before.^4^ The metagene signature MUCsig was calculated by taking the mean expression level of all genes present in the mucin family, scaled to a standard deviation of one and centered around zero. The publicly available murine data set derived from normal breast of pregnant mice (GEO ID: GSE8191^13^) was used to evaluate mucin expression in the normal breast during pregnancy. Ensembl database was used to convert mouse gene names to the human equivalent.

### Genome-wide copy number analysis

Hematoxylin and eosin slides from the archived FFPE blocks were reviewed by a pathologist (G.P.) to confirm diagnosis and evaluate tumor content. Samples with tumor purity below 60% were macrodissected (*N* = 56). DNA was extracted as described above. A total of 80 ng of DNA was used for copy number profiling using the Affymetrix OncoScan® FFPE Assay Kit according to the manufacturer’s instructions. The raw intensity values from the scanned chips were normalized to obtain Log2 ratios, B allele frequencies and genotyping calls (AA/AB/BB) using Affymetrix Power Tools. We used release NA.33 of the NetAffx library for the reference model and annotation. We computed the median absolute pairwise deviation and the median auto-correlation from the normalized log2 ratios as quality control metrics and used a threshold of 0.30 and 0.5, respectively, to flag failed arrays. Further details are provided in the Supplementary Methods.

### Library preparation and whole genome sequencing

For each of 53 patients, two samples of 1μg genomic DNA from tumor and histologically normal axillary lymph nodes were whole genome sequenced at The McDonnell Genome Institute at Washington University (St Louis MO, USA) on an Illumina HiSeqX platform. Briefly, manual dual indexed libraries were constructed with 1μg of FFPE genomic DNA for the 53 tumor/normal pairs using the Accel-NGS 2S Plus Library Kit (Swift, MI, USA). Samples were fragmented on the Covaris LE220 instrument with 350bp target insert size. PCR cycle optimization was performed to prevent over-amplification of the libraries. The concentration of each library was determined through qPCR (Kapa Biosystems, MA, USA). For the normal samples, each library was loaded on one lane of a HiSeqX flow cell, whereas for tumor samples, each library was loaded across two lanes of a HiSeqX flow cell. 2×150 paired-end sequence data were generated at a target depth of 30×(normal) and 60(tumor) haploid genome coverage. All sequencing data are available in EGA under accession “EGAS00001002685”. Further details are provided in the Supplementary Methods.

### Tumor heterogeneity

To quantify the level of intra-tumor heterogeneity present in a sample, we used the MATH score as previously described^9^;

$$MATH = \frac{{{\mathrm{MAD}}\left( {VAFs} \right)}}{{{\mathrm{median}}\left( {VAFs} \right)}}$$

where MAD(VAFs) is the median absolute deviation of the variant allele fractions (VAFs) of all the mutations (coding and noncoding) in a tumor.

### Mutational signature

All samples were analyzed using deconstructSigs^26^ to extract signatures based on the Wellcome Trust Sanger Institute Mutational Signature Framework.

### Statistical analysis and survival analysis

Except for age and date of diagnosis that were considered as continuous variables and therefore compared using the non-parametric Mann–Whitney *U* test, differences in other clinicopathological characteristics between BCP and controls were analyzed using the *χ*^2^ test or the Fisher exact test when appropriate. All statistical tests comparing BCP and controls were done using the non-parametric Mann–Whitney *U* test and the Fisher exact test for continuous and categorical variables, respectively. Independent association between continuous and binary variables with BCP vs. controls was investigated using linear and logistic regressions, respectively. All multivariate tests were adjusted for age at diagnosis, date of diagnosis, pathological stage, and molecular subtypes by IHC. All interaction and multivariate tests were done using analysis of variance to compare the models with and without the extra term.

All correlations were measured using the non-parametric Spearman’s *rho* coefficient. Reported *P-*values were two-tailed, and differences were considered significant when the *P*-value was less than 0.05. When applicable, multiple testing correction was done using the false discovery rate method (FDR),^27^ FDR below 0.05 being considered significant. All analyses were done in R software version 3.3.2 (available at www.r-project.org) and Bioconductor version 3.4.

Survival endpoint was DFS and calculated from the date of surgery to any loco-regional or distant recurrence, contralateral BC, other primary tumor or death from any cause, whichever occurred first. In the absence of any of the above-mentioned events, survival was censored at the last follow-up visit or phone call with the patient. Survival curves were estimated using the Kaplan–Meier method and compared by the log-rank test. The prognostic impact of pregnancy on survival was evaluated using univariate and multivariate Cox proportional hazards regression models and expressed as hazard ratio (HR) with 95% CI. Multivariate analysis was adjusted for standard clinical prognostic factors (age at diagnosis, date of diagnosis, pathological stage, and molecular subtypes by IHC). Further details are provided in the Supplementary Methods.

### Data availability

Raw gene expression data, together with patients’ characteristics, are publicly available on GEO http://www.ncbi.nlm.nih.gov/geo/, under accession number GSE53031. Sequencing data have been deposited at the European Genome-Phenome Archive (http://www.ebi.ac.uk/ega/), under accession number EGAS00001002685.

## Electronic supplementary material

Supplementary Information

Supplementary Table S1

Supplementary Table S4

Supplementary Table S3

## Electronic supplementary material

**Supplementary information** accompanies the paper on the *npj Breast Cancer* website (10.1038/s41523-018-0077-3).
