# Supplementary material for: Breast cancer diagnosed during pregnancy is associated with enrichment of non-silent mutations, mismatch repair deficiency signature and mucin mutations
Source: NPJ Breast Cancer. 2018 Aug 6;4:23. doi: 10.1038/s41523-018-0077-3 (PMC6078984; doi:10.1038/s41523-018-0077-3)
Supplement: Supplementary file 1 — Supplementary Information [file 41523_2018_77_MOESM1_ESM.pdf]

Supplemental Information

Breast cancer diagnosed during pregnancy is associated with enrichment of non-silent mutations, mismatch repair deficiency signature and mucin mutations

Bastien Nguyen, David Venet, Hatem A. Azim Jr, David Brown, Christine Desmedt, Matteo Lambertini, Samira Majjaj, Giancarlo Pruneri, Fedro Peccatori, Martine Piccart, Françoise Rothé and Christos Sotiriou

Supplementary Figures:

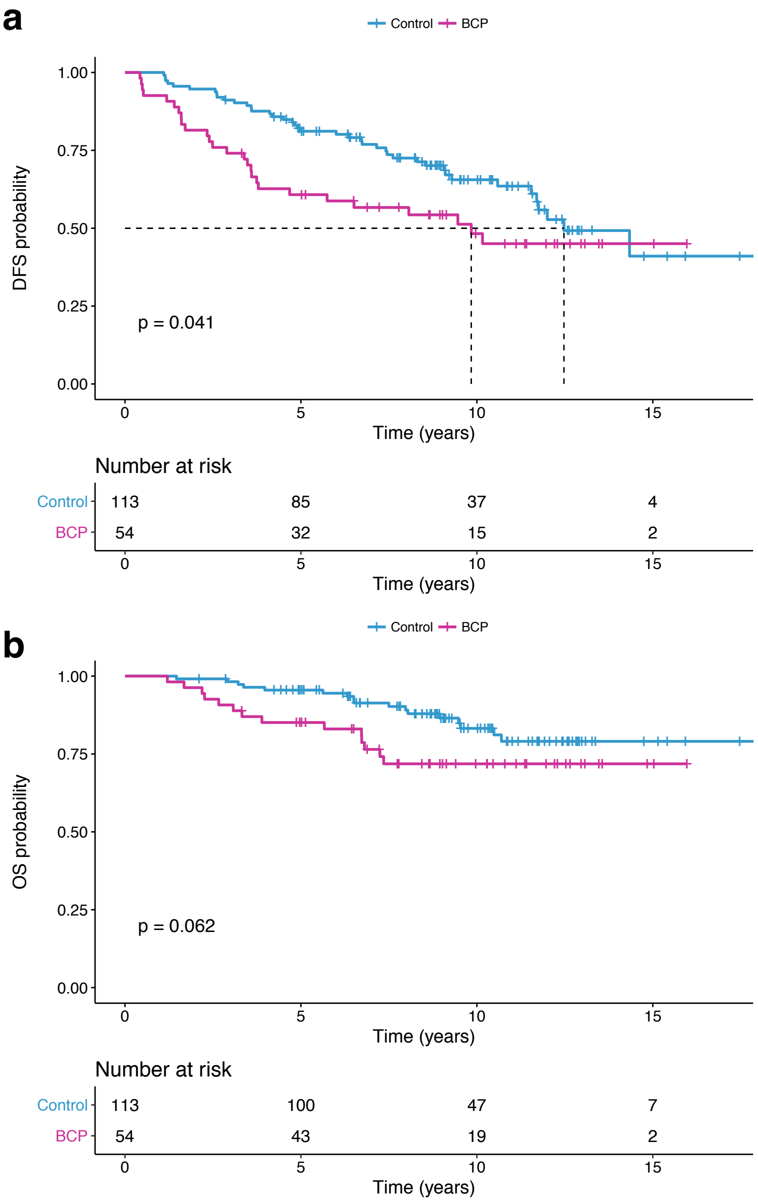

Supplementary Fig. S1. Updated survival analysis of BCP and controls. a Kaplan-Meier plot showing the DFS probability between control and BCP. **b** Kaplan-Meier plot showing the OS probability between control and BCP

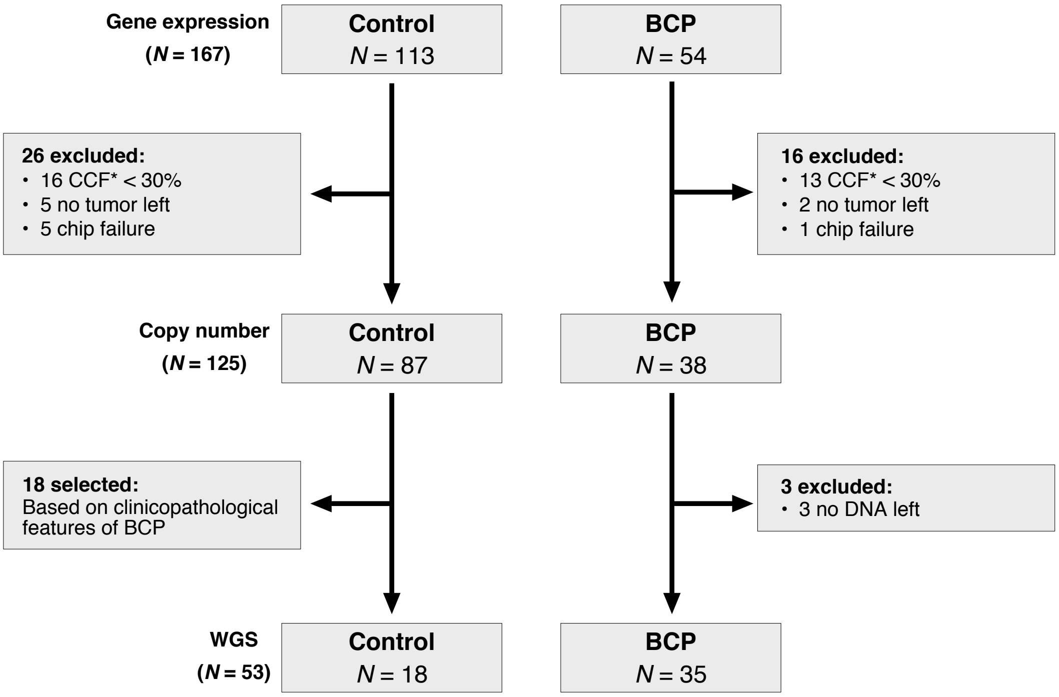

Supplementary Fig. S2. Flow chart summarizing the number of patients included in the analyses and the reasons for inclusion and exclusion. There were 167 patients with available gene expression profiles in the original cohort. Seven patients were excluded because the tumor FFPE blocks were exhausted. We performed genome-wide copy number profiling on a total of 160 patients comprising 108 breast cancer controls and 52 breast cancers diagnosed during pregnancy (BCP). After quality control, a total of 125 patients were analyzed. Whole genome sequencing (WGS) was performed on 53 matched normal and tumor samples, 35 of which were BCP.

*CCF, Cancer cell fraction estimated with the Genome Alteration Print algorithm.

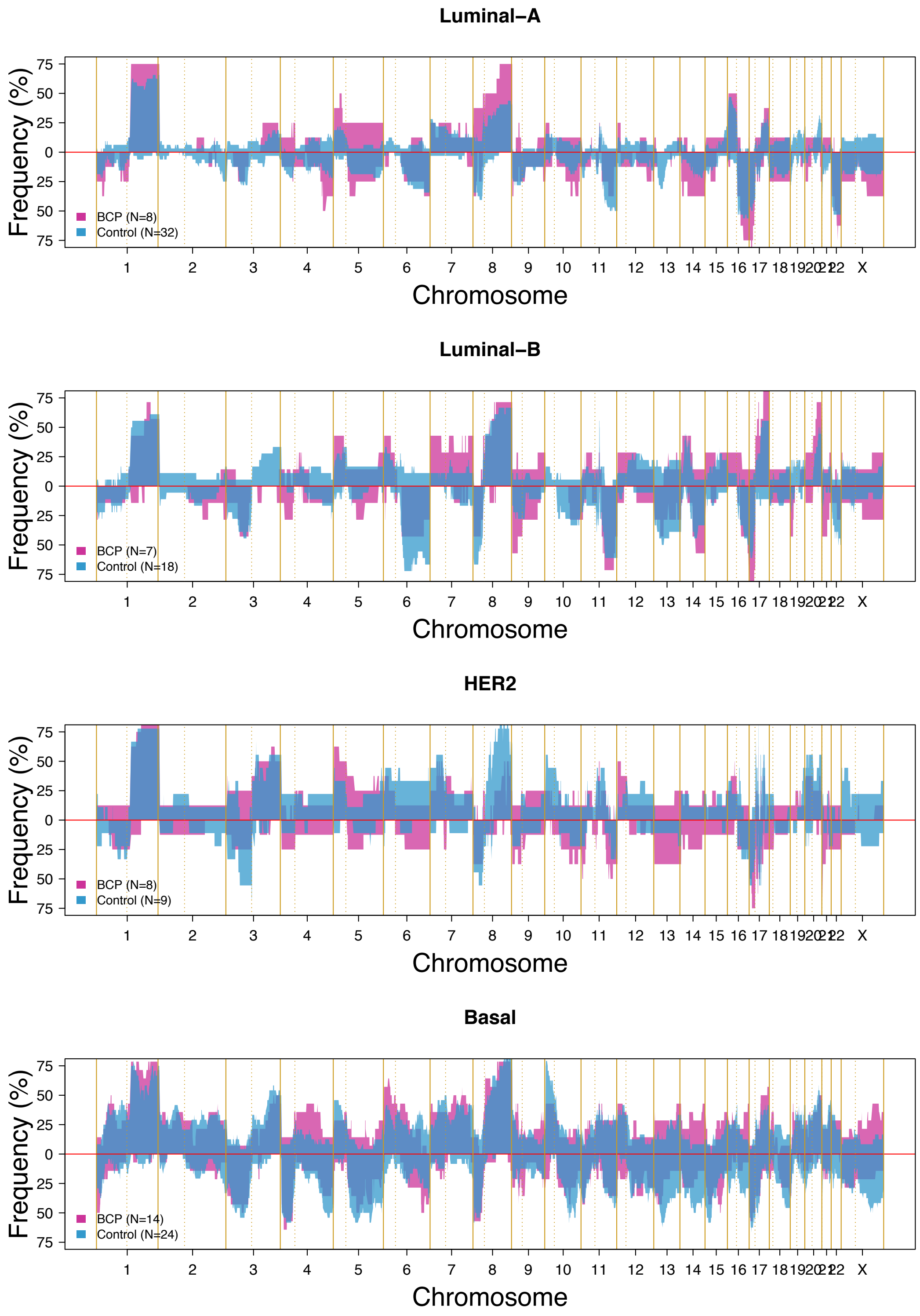

Supplementary Fig. S3. Comparison of the CNA frequencies of controls (blue) and BCP (pink) by intrinsic subtypes as defined by PAM50.

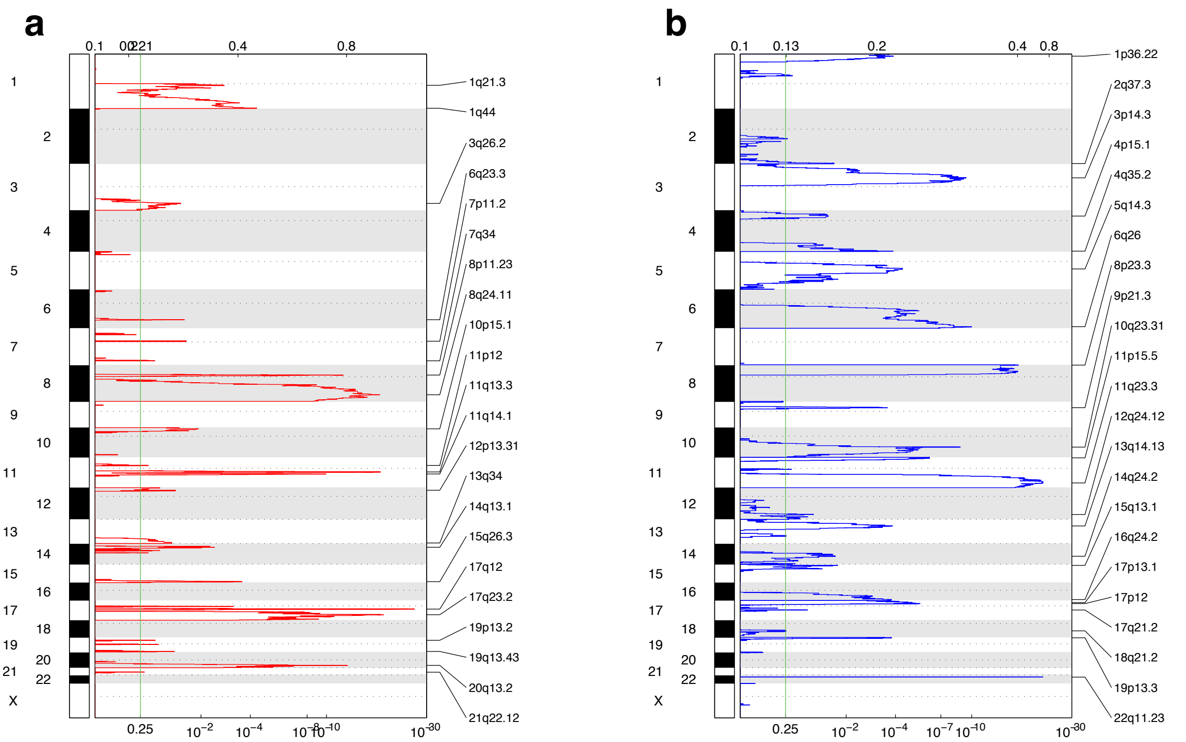

Supplementary Fig. S4. Genomic identification of significant targets in cancer (GISTIC) analysis. a GISTIC plot showing 22 amplification peaks. **b** GISTIC plot showing 23 deletion peaks.

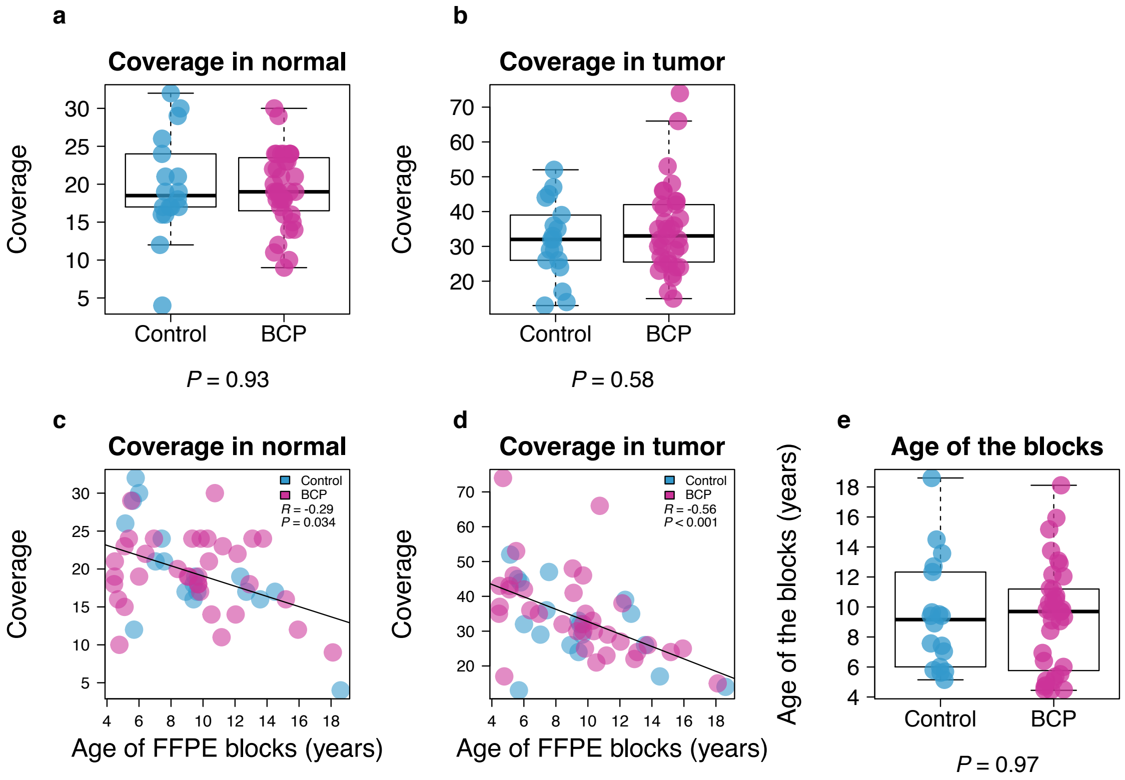

Supplementary Fig. S5. Coverage and age of the FFPE blocks. a-b Comparison of average coverage between BCP and controls in normal **(a)** and in tumor **(b)**. **c-d** Correlation between average coverage and the age of the blocks (years) in normal **(c)** and tumor **(d)**. **e** Comparison of the age of the blocks between controls and BCP.

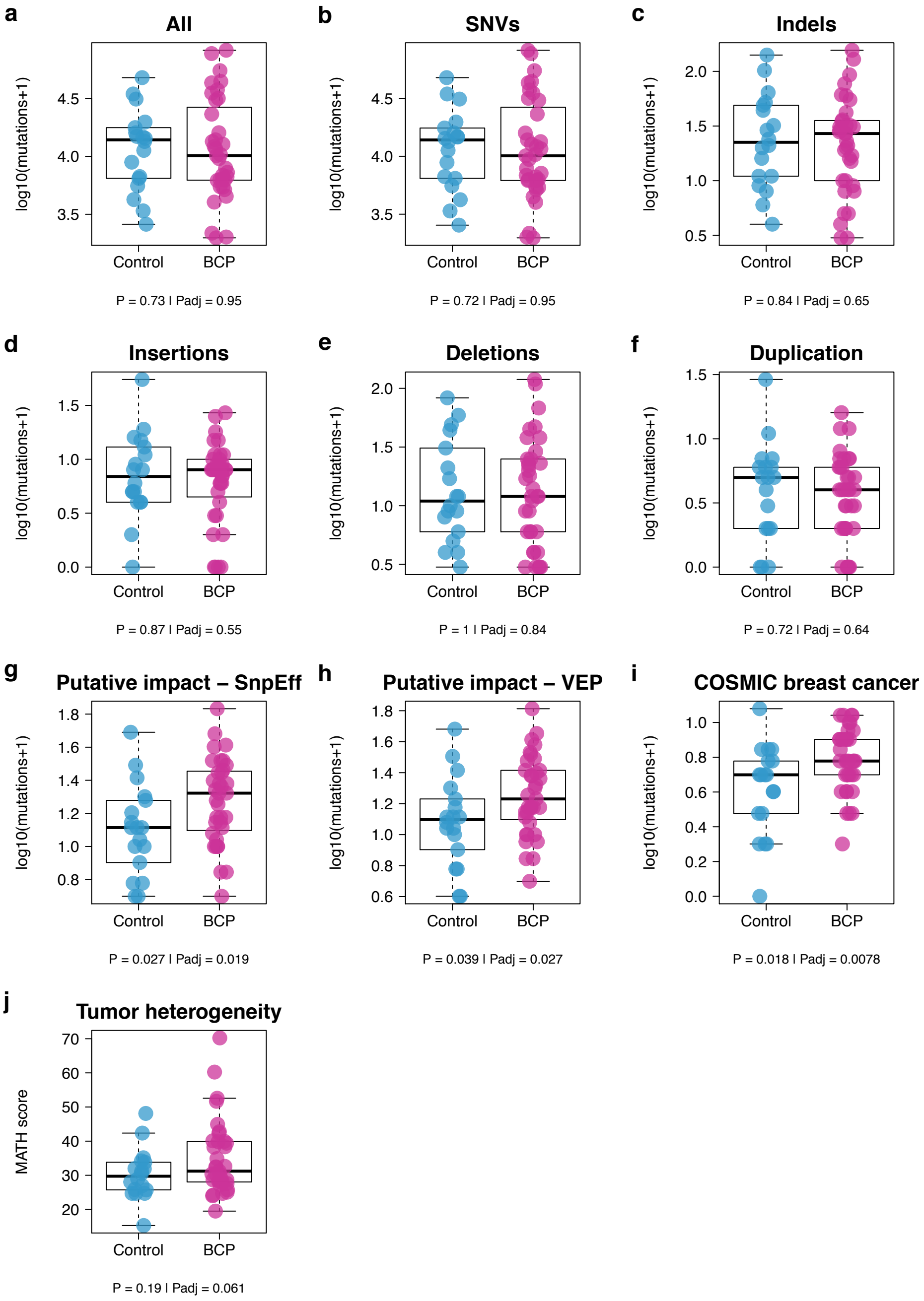

Supplementary Fig. S6. Mutational load and tumor heterogeneity. a-i Comparison of mutational load according to mutation types between controls and BCP. **j** Comparison of tumor heterogeneity as assessed by the MATH score between controls and BCP. *P*, *P*-value comparing BCP and controls using the non-parametric Mann–Whitney *U* test; P_{adj}, *P*-value adjusted for age at diagnosis, date of diagnosis, pathological stage and molecular subtypes by IHC using a linear regression.

a

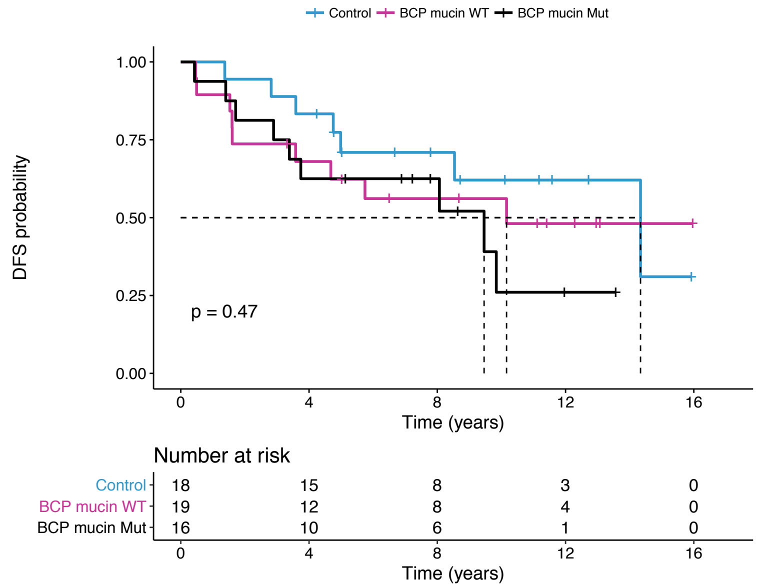

b

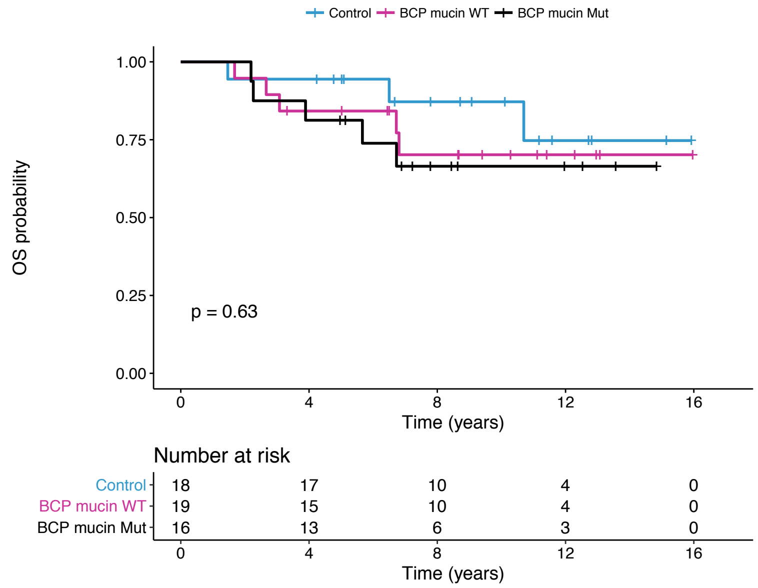

Supplementary Fig. S7. Survival analysis of controls and BCP according to mutational status of mucins. a Kaplan-Meier plot showing the DFS probability between control and BCP WT (pink) or mutated (black) for mucin. **b** Kaplan-Meier plot showing the OS probability between control and BCP WT (pink) or mutated (black) for mucin. p; log rank p-value comparing the three groups.

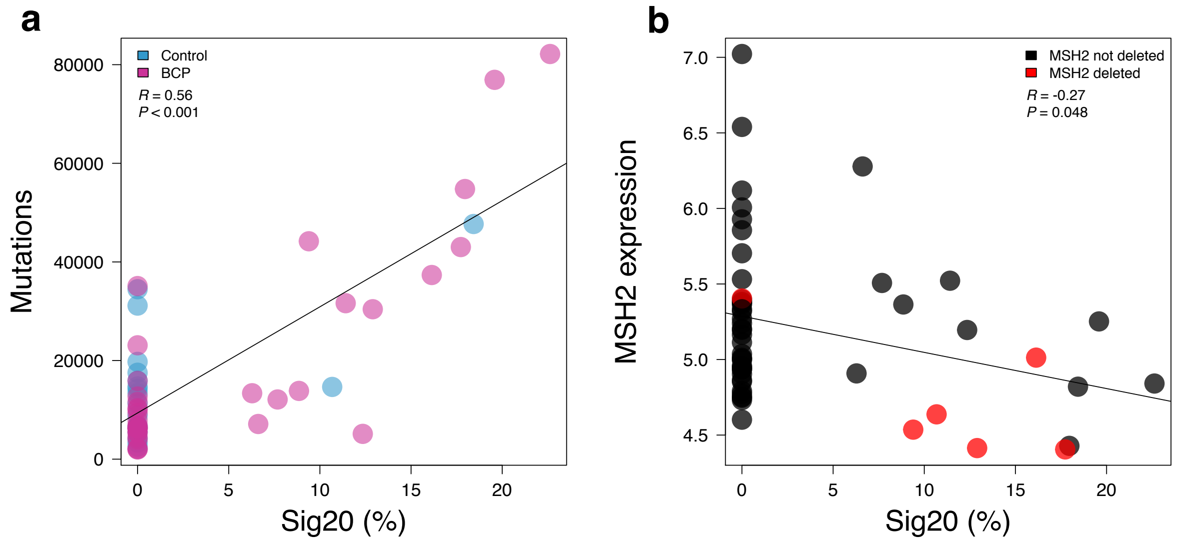

Supplementary Fig. S8. Relationship between SNV mutational load and MSH2 expression with signature 20 (Sig20) frequency. a Correlation between SNV mutational load and signature 20 frequency. **b** Correlation between MSH2 expression and signature 20 frequency.

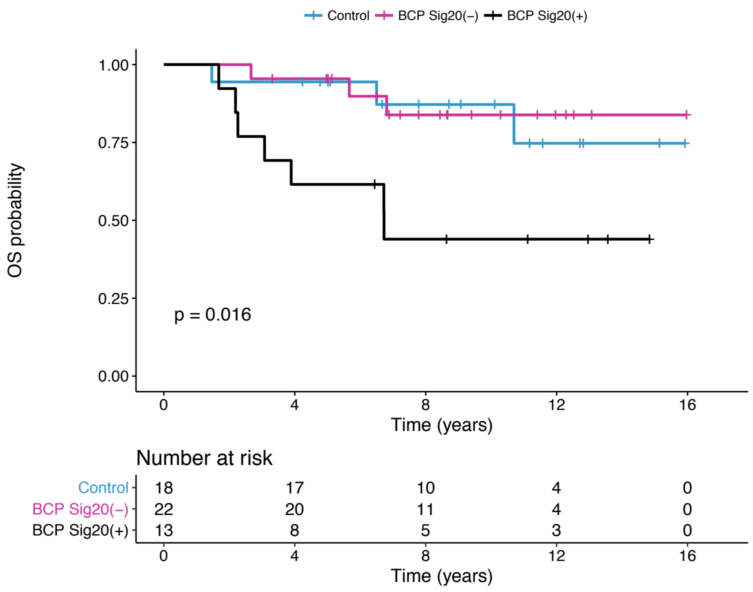

Supplementary Fig. S9. Survival analysis of BCP and controls. Kaplan-Meier plot showing the OS probability between control and BCP with (black) or without (pink) signature 20. p; log rank p-value comparing the three groups.

Supplementary Tables:

Supplementary Table S1. Individual patients data with complete clinicopathological features. (Provided as an individual CSV file)

Supplementary Table S2. Clinicopathological features of control and BCP patients included in the different analyses.

		Gene expression (N=167)			CNAs (N=125)			WGS (N=53)		
		Control	BCP	P	Control	BCP	P	Control	BCP	P
N		113	54		87	38		18	35	
Median DFS time (years)		12.47	9.84	0.041[a]	12	9.46	0.112[a]	14.34	9.46	0.289[a]
5-year OS rate (95% CI)		95.5% (91.7% - 99.4%)	85.1% (76.1% - 95.2%)		94.2% (89.4% - 99.3%)	81.4% (69.9% - 94.8%)		94.4% (84.4% - 100%)	82.8% (71.1% - 96.3%)	
Age at diagnosis	Median years (range)	36 (28-47)	36 (28-47)	0.677[b]	36 (28-47)	36 (29-44)	0.796[b]	35 (30-43)	36 (29-44)	0.202[b]
Date of diagnosis	Median years (range)	2005 (1996-2009)	2005 (1996-2010)	0.743[b]	2005 (1996-2009)	2005 (1996-2010)	0.776[b]	2005 (1996-2009)	2005 (1996-2010)	0.97[b]
Gestational age at diagnosis (trimester)	First		26 (48.1%)			18 (47.4%)			17 (48.6%)	
	Second		15 (27.8%)			11 (28.9%)			11 (31.4%)	
	Third		13 (24.1%)			9 (23.7%)			7 (20%)	
Stage	I	27 (24.3%)	14 (26.4%)		19 (22.1%)	7 (18.4%)		2 (11.1%)	7 (20%)	
	II	55 (49.5%)	27 (50.9%)		47 (54.7%)	21 (55.3%)		12 (66.7%)	20 (57.1%)	
	III	29 (26.1%)	12 (22.6%)	0.88	20 (23.3%)	10 (26.3%)	0.87	4 (22.2%)	8 (22.9%)	0.79
Tumor size	<= 2cm	62 (56.9%)	28 (53.8%)		52 (61.2%)	24 (63.2%)		12 (66.7%)	21 (60%)	
	> 2cm	47 (43.1%)	24 (46.2%)	0.85	33 (38.8%)	14 (36.8%)	0.99	6 (33.3%)	14 (40%)	0.86
Nodal status	Negative	52 (46%)	26 (48.1%)		40 (46%)	18 (47.4%)		8 (44.4%)	17 (48.6%)	
	Positive	61 (54%)	28 (51.9%)	0.93	47 (54%)	20 (52.6%)	1	10 (55.6%)	18 (51.4%)	1
Grade	1	4 (3.7%)	3 (5.8%)		3 (3.5%)	3 (7.9%)		2 (11.1%)	3 (8.6%)	
	2	44 (40.4%)	17 (32.7%)		31 (36.5%)	9 (23.7%)		4 (22.2%)	9 (25.7%)	
	3	61 (56%)	32 (61.5%)	0.56	51 (60%)	26 (68.4%)	0.24	12 (66.7%)	23 (65.7%)	1
ER	Positive	88 (77.9%)	36 (66.7%)		67 (77%)	23 (60.5%)		13 (72.2%)	22 (62.9%)	
	Negative	25 (22.1%)	18 (33.3%)	0.17	20 (23%)	15 (39.5%)	0.095	5 (27.8%)	13 (37.1%)	0.71
PR	Positive	79 (69.9%)	35 (64.8%)		62 (71.3%)	22 (57.9%)		12 (66.7%)	20 (57.1%)	
	Negative	34 (30.1%)	19 (35.2%)	0.63	25 (28.7%)	16 (42.1%)	0.21	6 (33.3%)	15 (42.9%)	0.71
HER2	Negative	91 (80.5%)	45 (83.3%)		71 (81.6%)	31 (81.6%)		11 (61.1%)	29 (82.9%)	
	Positive	22 (19.5%)	9 (16.7%)	0.82	16 (18.4%)	7 (18.4%)	1	7 (38.9%)	6 (17.1%)	0.1
Molecular subtypes by IHC	Lum A-like	28 (24.8%)	15 (27.8%)		21 (24.1%)	9 (23.7%)		3 (16.7%)	9 (25.7%)	
	Lum B-like	42 (37.2%)	18 (33.3%)		33 (37.9%)	12 (31.6%)		5 (27.8%)	11 (31.4%)	
	Lum HER2	18 (15.9%)	5 (9.3%)		13 (14.9%)	4 (10.5%)		5 (27.8%)	3 (8.6%)	
	ERneg HER2-like	4 (3.5%)	4 (7.4%)		3 (3.4%)	3 (7.9%)		2 (11.1%)	3 (8.6%)	
	TNBC	21 (18.6%)	12 (22.2%)	0.58	17 (19.5%)	10 (26.3%)	0.66	3 (16.7%)	9 (25.7%)	0.45
PAM50	Luminal-A	40 (35.4%)	13 (24.1%)		32 (36.8%)	8 (21.1%)		3 (16.7%)	8 (22.9%)	
	Luminal-B	23 (20.4%)	10 (18.5%)		18 (20.7%)	7 (18.4%)		4 (22.2%)	7 (20%)	
	HER2	12 (10.6%)	10 (18.5%)		9 (10.3%)	8 (21.1%)		5 (27.8%)	8 (22.9%)	
	Basal	32 (28.3%)	18 (33.3%)		24 (27.6%)	14 (36.8%)		5 (27.8%)	11 (31.4%)	
	Normal-like	6 (5.3%)	3 (5.6%)	0.45	4 (4.6%)	1 (2.6%)	0.25	1 (5.6%)	1 (2.9%)	0.96

CNAs, copy number alterations; WGS, whole genome sequencing; BCP, breast cancer diagnosed during pregnancy; ER, Estrogen receptor; PR, Progesterone receptor; IHC, Immunohistochemistry; P,

p-value derived from $\chi 2$ test or the Fisher exact test when appropriate ([a]except DFS derived from the logank test and [b]continuous variable derived from Mann–Whitney *U* test)

Supplementary Table S3. Results from the CNAs analysis by segments comparing BCP and control and by molecular subgroups. (Provided as an individual CSV file)

Supplementary Table S4. Catalogues of every non-silent mutations found in the whole cohort and annotated by SnpEff and VEP.

Supplementary Table S5. Clinicopathological features between control, BCP without mucins mutations, and BCP with mucins mutations.

		Control Mucin WT	Control Mucin mut	P		BCP Mucin WT	BCP Mucin mut	P
N		16	2			19	16	
Age at diagnosis	Median years (range)	35 (30-40)	42.5 (42-43)	0.0275[a]		37 (30-44)	36 (29-41)	0.894[a]
Stage	I	1 (6.2%)	1 (50%)			4 (21.1%)	3 (18.8%)	
	II	11 (68.8%)	1 (50%)			11 (57.9%)	9 (56.2%)	
	III	4 (25%)	0 (0%)	0.25		4 (21.1%)	4 (25%)	1
Tumor size	<= 2cm	11 (68.8%)	1 (50%)			11 (57.9%)	10 (62.5%)	
	> 2cm	5 (31.2%)	1 (50%)	1		8 (42.1%)	6 (37.5%)	1
Nodal status	Negative	6 (37.5%)	2 (100%)			10 (52.6%)	7 (43.8%)	
	Positive	10 (62.5%)	0 (0%)	0.18		9 (47.4%)	9 (56.2%)	0.85
Grade	1	1 (6.2%)	1 (50%)			2 (10.5%)	1 (6.2%)	
	2	3 (18.8%)	1 (50%)			5 (26.3%)	4 (25%)	
	3	12 (75%)	0 (0%)	0.098		12 (63.2%)	11 (68.8%)	1
ER	Positive	11 (68.8%)	2 (100%)			12 (63.2%)	10 (62.5%)	
	Negative	5 (31.2%)	0 (0%)	1		7 (36.8%)	6 (37.5%)	1
PR	Positive	10 (62.5%)	2 (100%)			12 (63.2%)	8 (50%)	
	Negative	6 (37.5%)	0 (0%)	0.53		7 (36.8%)	8 (50%)	0.66
HER2	Negative	9 (56.2%)	2 (100%)			17 (89.5%)	12 (75%)	
	Positive	7 (43.8%)	0 (0%)	0.5		2 (10.5%)	4 (25%)	0.38
Molecular subtypes	Lum A-like	2 (12.5%)	1 (50%)			5 (26.3%)	4 (25%)	
	Lum B-like	4 (25%)	1 (50%)			7 (36.8%)	4 (25%)	
	Lum HER2	5 (31.2%)	0 (0%)			1 (5.3%)	2 (12.5%)	
	ERneg HER2-like	2 (12.5%)	0 (0%)			1 (5.3%)	2 (12.5%)	
	TNBC	3 (18.8%)	0 (0%)	0.84		5 (26.3%)	4 (25%)	0.92
PAM50	Luminal-A	2 (12.5%)	1 (50%)			6 (31.6%)	2 (12.5%)	
	Luminal-B	4 (25%)	0 (0%)			2 (10.5%)	5 (31.2%)	
	HER2	5 (31.2%)	0 (0%)			4 (21.1%)	4 (25%)	
	Basal	5 (31.2%)	0 (0%)			6 (31.6%)	5 (31.2%)	
	Normal-like	0 (0%)	1 (50%)	0.039		1 (5.3%)	0 (0%)	0.42

BCP, breast cancer diagnosed during pregnancy; ER, Estrogen receptor; PR, Progesterone receptor; IHC, Immunohistochemistry; P, p-value derived from $\chi 2$ test or the Fisher exact test when appropriate ([a]except continuous variable derived from Mann–Whitney *U* test)

Supplementary Table S6. Clinicopathological features between control, BCP Sig20-negative patients, and BCP Sig20-positive patients.

		Control Sig20-	Control Sig20+	P		BCP Sig20-	BCP Sig20+	P
**N**		**16**	**2**			**22**	**13**	
**Age at diagnosis**	**Median years (range)**	35 (30-43)	34.5 (33-36)	0.887[a]		36 (30-44)	37 (29-41)	0.38[a]
**Stage**	**I**	1 (6.2%)	1 (50%)			5 (22.7%)	2 (15.4%)	
	**II**	11 (68.8%)	1 (50%)			13 (59.1%)	7 (53.8%)	
	**III**	4 (25%)	0 (0%)	0.25		4 (18.2%)	4 (30.8%)	0.71
**Tumor size**	**<= 2cm**	11 (68.8%)	1 (50%)			13 (59.1%)	8 (61.5%)	
	**> 2cm**	5 (31.2%)	1 (50%)	1		9 (40.9%)	5 (38.5%)	1
**Nodal status**	**Negative**	6 (37.5%)	2 (100%)			11 (50%)	6 (46.2%)	
	**Positive**	10 (62.5%)	0 (0%)	0.18		11 (50%)	7 (53.8%)	1
**Grade**	**1**	2 (12.5%)	0 (0%)			2 (9.1%)	1 (7.7%)	
	**2**	4 (25%)	0 (0%)			7 (31.8%)	2 (15.4%)	
	**3**	10 (62.5%)	2 (100%)	1		13 (59.1%)	10 (76.9%)	0.73
**ER**	**Positive**	11 (68.8%)	2 (100%)			16 (72.7%)	6 (46.2%)	
	**Negative**	5 (31.2%)	0 (0%)	1		6 (27.3%)	7 (53.8%)	0.16
**PR**	**Positive**	10 (62.5%)	2 (100%)			16 (72.7%)	4 (30.8%)	
	**Negative**	6 (37.5%)	0 (0%)	0.53		6 (27.3%)	9 (69.2%)	0.038
**HER2**	**Negative**	11 (68.8%)	0 (0%)			20 (90.9%)	9 (69.2%)	
	**Positive**	5 (31.2%)	2 (100%)	0.14		2 (9.1%)	4 (30.8%)	0.17
**Molecular subtypes**	**Lum A-like**	3 (18.8%)	0 (0%)			8 (36.4%)	1 (7.7%)	
	**Lum B-like**	5 (31.2%)	0 (0%)			7 (31.8%)	4 (30.8%)	
	**Lum HER2**	3 (18.8%)	2 (100%)			2 (9.1%)	1 (7.7%)	
	**ERneg HER2-like**	2 (12.5%)	0 (0%)			0 (0%)	3 (23.1%)	
	**TNBC**	3 (18.8%)	0 (0%)	0.44		5 (22.7%)	4 (30.8%)	0.091
**PAM50**	**Luminal-A**	3 (18.8%)	0 (0%)			7 (31.8%)	1 (7.7%)	
	**Luminal-B**	4 (25%)	0 (0%)			5 (22.7%)	2 (15.4%)	
	**HER2**	3 (18.8%)	2 (100%)			3 (13.6%)	5 (38.5%)	
	**Basal**	5 (31.2%)	0 (0%)			6 (27.3%)	5 (38.5%)	
	**Normal-like**	1 (6.2%)	0 (0%)	0.3		1 (4.5%)	0 (0%)	0.24

BCP, breast cancer diagnosed during pregnancy; ER, Estrogen receptor; PR, Progesterone receptor; IHC, Immunohistochemistry; P, p-value derived from $\chi 2$ test or the Fisher exact test when appropriate ([a]except continuous variable derived from Mann–Whitney *U* test)

Supplementary Table S7. Clinicopathological features of BCP and TCGA controls.

		BCP	TCGA controls	P
**N**		**35**	**56**	
**Age at diagnosis**	**Median years (range)**	36.5 (30-39)	36 (29-44)	0.957[a]
**Date of diagnosis**	**Median years (range)**	2007 (1995-2011)	2005 (1996-2010)	0.132[a]
**Tumor size**	**<= 2cm**	21 (60%)	45 (80.4%)	
	**> 2cm**	14 (40%)	11 (19.6%)	0.061
**Nodal status**	**Negative**	17 (48.6%)	21 (37.5%)	
	**Positive**	18 (51.4%)	35 (62.5%)	0.41
**ER status**	**Negative**	13 (37.1%)	16 (28.6%)	
	**Positive**	22 (62.9%)	40 (71.4%)	0.53
**PR status**	**Negative**	15 (42.9%)	16 (28.6%)	
	**Positive**	20 (57.1%)	40 (71.4%)	0.24
**HER2 status**	**Negative**	29 (82.9%)	48 (85.7%)	
	**Positive**	6 (17.1%)	8 (14.3%)	0.95

BCP, breast cancer diagnosed during pregnancy; ER, Estrogen receptor; PR, Progesterone receptor; P, p-value derived from $\chi 2$ test or the Fisher exact test when appropriate ([a]except continuous variable derived from Mann–Whitney *U* test)

Supplementary Methods:

<u>Genome-wide copy number analysis</u>

We used two parallel approaches involving (a) allele specific copy number analysis using heterozygous SNP probes and (b) total copy number analysis using the full set of 200 K markers and parameters from (a) to control for the cancer cell fraction (CCF) and genomic mass. From the BAF and genotyping calls, only informative SNP probes displaying heterozygous genotype (AB) and 0.1 < BAF < 0.9 were kept for analysis at (a). The Log2 ratios and BAF were smoothed using the median absolute deviation and segmented jointly using a multitrack segmentation algorithm from the library copynumber[1] to determine common breakpoints. Estimates of CCF and genomic mass were obtained using GAP[2]. Samples with a CCF lower than 30% were further excluded. For analysis at (b), the Log2 ratios for the same samples analyzed at (a) were segmented by penalized least square regression as above and non-rounded estimates of copy numbers y were obtained as

$$y = \frac{1}{a}\left(2^{\frac{x}{c}}\big(\psi a + 2(1-a)\big) - 2(1-a)\right)$$

where a is the CCF and ψ is the genomic mass, both estimated at (a). c = 0.8 is a constant representing the compression ratio of the array and finally x is the observed Log2 ratio of a given segment. The copy numbers were categorized as deletions (−1) if $y < \psi - 0.5$, gains (+1) if $y > \psi + 0.5$, amplifications (+2) if $y > \psi + 2.5$, and copy neutral (0) otherwise. Unless otherwise stated, all parameter settings were kept at default values and all computations were done using R/Bioconductor. Segmented data were used as input for Genomic Identification of Significant Targets in Cancer, version 2.0 (GISTIC 2.0)[3] and version 6.2 on the Broad Institute GenePattern cloud server to obtain somatic focal and broad CNA events. These were then parsed in R. For focal events, only "high-level" focal amplification events, defined as log2 ratio >0.9 were retained, whereas focal losses were retained with log2 ratio >0.3 and with a Q value <0.25.

Broad events, defined as arm-level events encompassing 98% or more of a chromosome arm, were computed using GISTIC as well. For gene-levels analysis, we also used the gene level output given by GISTIC analysis. Unless otherwise stated, all parameter settings were kept at default values and all computations were done using R/Bioconductor.

Sequence alignment and variant calling

Adapters were trimmed using Trimmomatic[4]. Paired sequence reads were aligned to reference human genome build hg19/GRCh37 using bwa mem[5]. Marking and removal of duplicates were done using biobambam[6] while bases in overlapping reads from the same read pair were removed with BamUtil clipOverlap[7]. Somatic mutations were called using Strelka[8]. Except for mutations identified in the COSMIC database[9] related to breast cancer, mutations were filtered using default quality thresholds with a QSS_NT > 15 for SNVs and a QSI_NT> 30 for Indels. We also filtered out SNVs when <5 sequence reads reported a variant allele in the tumor. Variant annotation and effect prediction were carried out using SnpEff (v. 4.3p)[10] and Ensembl Variant Effect Predictor (VEP)[11]. Mutations with putative impact were defined as those assigned a high or moderate impact from SnpEff. To predict the pathogenicity of non-synonymous SNVs we also used a battery of in silico algorithms (SIFT, PolyPhen and ConDel) [12–14].

Significance of the missense mutation in mucins producing a serine

Because (i) the distribution of bases is not uniform, (ii) the distribution of the different missense mutations is not uniform, and (iii) the distribution of bases within each mucin varies, we used an empirical approach to test the significance of the missense mutation in mucins producing a serine. First, we defined the probability of mutation at a base b (b in A, C, G, T) P_b:

$$P_b = \frac{P_b^n}{P_b^g}$$

where P_b^n is the proportion of the bases *b* that are affected by a missense mutation in the whole cohort and P_b^g is the proportion of the base *b* in the coding bases of the whole genome. For each mucin, we determine P_{muc} as the probability of having a mutation in base *b* in a mucin *muc*:

$$P_b^{muc} = \frac{\left(P_b \cdot \acute{P}_b^{\mathrm{muc}}\right)}{\sum_{b'}\left(P_{b'} \cdot \acute{P}_{b'}^{\mathrm{muc}}\right)}$$

where P_b is the baseline probability above-mentioned and $\acute{P}_b^{\mathrm{muc}}$ is the proportion of the coding bases present in mucin muc. Using P_b^{muc} we artificially generated 1000 random missense mutations in each mucin and calculated the proportion of altered codon producing a serine. For each random mutation, we first drawn randomly the type of base mutated base on P_b^{muc}. The resulting nucleotide was randomly chosen based on the observed mutations. The precise nucleotide mutated among those corresponding to the base mutated in the mucin was chosen randomly. If the resulting mutation did not lead to a missense mutation it was discarded. The number of serine obtained after 1000 random mutations was used to derive a probability to obtain a serine by the play of chance in each mucin: P_{muc}^{ser}.

To estimate the probability of observing *N* serines in total in the mucin mutated, we again used a Monte-Carlo method. We simulated 10^5 scenarios, each with the same mucin mutated as in the real dataset, and drawn randomly whether each mutation led to a serine, using P_{muc}^{ser}.

Finally, the empirical p-value was calculated by using the Monte-Carlo procedure[15]

$$p = \frac{(r+1)}{(n+1)}$$

where *r* is the number of simulations that produced at least *N* serines and *n* is the number simulations (source code available upon request).

References

1. Nilsen, G. *et al.* Copynumber: Efficient algorithms for single- and multi-track copy number segmentation. *BMC Genomics* **13,** 591 (2012).
2. Popova, T. *et al.* Genome Alteration Print (GAP): a tool to visualize and mine complex cancer genomic profiles obtained by SNP arrays. *Genome Biol.* **10,** R128 (2009).
3. Mermel, C. H. *et al.* GISTIC2.0 facilitates sensitive and confident localization of the targets of focal somatic copy-number alteration in human cancers. *Genome Biol.* **12,** R41 (2011).
4. Bolger, A. M., Lohse, M. & Usadel, B. Trimmomatic: a flexible trimmer for Illumina sequence data. *Bioinformatics* **30,** 2114–2120 (2014).
5. Li, H. & Durbin, R. Fast and accurate short read alignment with Burrows-Wheeler transform. *Bioinformatics* **25,** 1754–1760 (2009).
6. Tischler, G. & Leonard, S. biobambam: tools for read pair collation based algorithms on BAM files. *Source Code Biol. Med.* **9,** 13 (2014).
7. http://genome.sph.umich.edu/wiki/BamUtil.
8. Saunders, C. T. *et al.* Strelka: accurate somatic small-variant calling from sequenced tumor–normal sample pairs. *Bioinformatics* **28,** 1811–1817 (2012).
9. Forbes, S. A. *et al.* COSMIC: Somatic cancer genetics at high-resolution. *Nucleic Acids Res.* **45,** D777–D783 (2017).
10. Zhang, D. *et al.* A program for annotating and predicting the effects of single nucleotide polymorphisms, SnpEff: SNPs in the genome of Drosophila melanogaster strain w1118 ; iso-2; iso-3. *Fly (Austin).* 321–329 (2012).
11. McLaren, W. *et al.* The Ensembl Variant Effect Predictor. *Genome Biol.* 042374 (2016). doi:10.1186/s13059-016-0974-4
12. González-Pérez, A. & López-Bigas, N. Improving the assessment of the outcome of

nonsynonymous SNVs with a consensus deleteriousness score, Condel. *Am. J. Hum. Genet.* **88,** 440–9 (2011).

13. Adzhubei, I. A. *et al.* A method and server for predicting damaging missense mutations. *Nat. Methods* **7,** 248–249 (2010).

14. Sim, N.-L. *et al.* SIFT web server: predicting effects of amino acid substitutions on proteins. *Nucleic Acids Res.* **40,** W452-7 (2012).

15. North, B. V, Curtis, D. & Sham, P. C. A note on the calculation of empirical P values from Monte Carlo procedures. *Am. J. Hum. Genet.* **71,** 439–41 (2002).
